# Book Review: Judgment, Decision-Making, and Embodied Choices

**DOI:** 10.3389/fpsyg.2021.665728

**Published:** 2021-04-06

**Authors:** Alex Miklashevsky, Elena Kulkova, Alexej Michirev, Melinda A. Mende, Matias Bertonatti

**Affiliations:** ^1^Potsdam Embodied Cognition Group, Cognitive Sciences, University of Potsdam, Potsdam, Germany; ^2^Department of Performance Psychology, Institute of Psychology, German Sport University Cologne, Cologne, Germany; ^3^Institut für Psychologie, Humboldt-Universität zu Berlin, Berlin, Germany

**Keywords:** embodied cognition, decision making, embodied choice, book review, mind-body

The book *Judgment, Decision-Making, and Embodied Choices* by Dr. Markus Raab, a cognitive psychologist working in the field of embodied cognition and sport sciences, is an engaging read. It discusses the role that our bodies play in our perception, decision making, and interaction with the world. The author expertly presents the theoretical underpinnings of embodied cognition theory and supports them with ample scientific evidence based on both well-known and recent research.

The book introduces the concept of embodied choices, those in which the sensorimotor system provides information to aid our decision making. This form of intuition, a “gut feeling,” opposes deliberate and rational decisions. While “gut feeling” sounds rather mysterious and the gut is even called “the second brain,” it relates here to fast-and-frugal heuristics. Spontaneous decisions based on gut feelings are important and work well even when they are made for the first time and one cannot rely on any prior experience with that specific choice, especially because embodied decisions (financial, medical, etc.) are made under high uncertainty and all parameters are almost never known.

The book has 13 chapters, which systematically disentangle the body-brain relationship. It progresses from basic research to more applied perspectives, such as decision making in sports or in everyday life, dealing with situations of high uncertainty (such as health or financial decisions), points out cultural and individual differences in decision making and even includes a chapter on training embodied choices. The book ends with an updated perspective of the uncertainty in the COVID-19 times and provides a take-home message on how we can implement these ideas to improve our everyday life. Discussions on recent topics such as COVID-19 and Brexit make the book up-to-date.

We think this book deserves your precious time because its author builds an inspiring bridge between decision making and embodiment by integrating scientific studies with personal experiences. He entertains us with personal storytelling and provides references for further reading. Giving a broad overview of up-to-date research in the field, asking intriguing questions, and providing thought-provoking real-life examples, the author honestly acknowledges that many questions remain open and more research is needed.

The book shares some features with Beilock's *How the Body Knows Its Mind: The Surprising Power of the Physical Environment to Influence How You Think and Feel*. Both explain captivatingly how the context, the environment in which people act, and their individual differences together affect numerous everyday choices and decisions, often unnoticed. A unique point is the profound description of the embodiment of sport decision making and performance. This area of research started attracting scientific attention recently, so a timely review from a prominent expert with an outline of future directions and practical implications is much appreciated. The structure could, however, be improved in future editions by adding summaries after each chapter.

The book is written in clear and understandable language, appropriate for a broad audience. While the content presents little surprise for experts, it is very useful for students and young scientists. The book provides a nicely structured and well-organized perspective on the theory of embodied cognition and offers captivating reading for non-experts, who will acquire a better understanding of the body-brain interconnections that can affect our everyday life. Coaches and teachers will derive inspirations to update their teaching methods by integrating the body more. The book may be appropriate for undergraduate-level teaching or can be used as additional course reading for students of cognitive science, psychology, sports sciences, and other disciplines focusing on decision making in various fields, including practical decisions in health, finance, or policy making, advertising, or ergonomics. However, the book is thought-provoking and does not give ready-to-use recipes.

While many students could benefit from getting familiar with the valuable overview of the interdisciplinary research presented in the book, its relatively high price makes the book hardly affordable for most students.

The author's style, with autobiographical descriptions opening the chapters, allows readers to meet the author as a person. He involves readers in his thought processes and highlights the idea of embodied choices even in the process of writing itself. These insertions serve a deeper function that becomes clear after having read the book. The author's extensive network (he is personally familiar with many of the researchers in the field) and his ability to put abstract ideas into an everyday context invite readers to think outside the box and connect research with their own practice. At the same time, a number of aspects could be improved, such as some of the graphic representations, which could communicate his ideas more clearly.

The book ends with “Ten statements for simplifying your life with embodied choices,” summarizing the most important ideas of the book. This chapter serves an important didactic purpose: *repetitio est mater studiorum*, repetition is the mother of learning. The rich potential of practical implications could be further explored in future editions by giving even more examples here, including those that are not covered in the book, thus once again stimulating readers' own curiosity and mental activity.

## Author Contributions

All authors listed have made a substantial, direct and intellectual contribution to the work, and approved it for publication.

## Conflict of Interest

AMic is a Ph.D. student of Prof. Dr. Markus Raab. The remaining authors declare that the research was conducted in the absence of any commercial or financial relationships that could be construed as a potential conflict of interest.

